# The pharmacologic basis of high dose chemotherapy with haematopoietic stem cell support for solid tumours

**DOI:** 10.1054/bjoc.2001.1970

**Published:** 2001-08

**Authors:** L F Porrata, A A Adjei

**Affiliations:** Division of Medical Oncology, Mayo Clinic, 200 First Street SW Rochester, MN, 55905, USA

**Keywords:** preclinical pharmacology, high-dose chemotherapy, solid tumours

## Abstract

The theoretical basis of high dose chemotherapy with haematopoietic stem cell support (HDT) for solid tumours is the presumption of a linear, steep dose-response relationship for chemotherapy conditioning agents. We review preclinical pharmacologic studies evaluating steep dose-response relationships for different chemotherapeutic agents, identified through a MED-LINE and CANCER-LIT search of the English medical literature from January 1966 to December 1999. Only BCNU, melphalan, nitrogen mustard, and the combination of thiotepa and cyclophosphamide demonstrated steep dose-response relationships over a wide dose-range. The pharmacologic evidence for the use of other antineoplastic agents for HDT in solid tumours is non-existent. More preclinical studies are needed for a rational development of this therapeutic approach for solid tumours. © 2001 Cancer Research Campaign http://www.bjcancer.com


*British Journal of Cancer* (2001) **85**(4), 484–489
© 2001 Cancer Research Campaign
doi: 10.1054/ bjoc.2001.1970, available online at http://www.idealibrary.com on IDEAL

http://www.bjcancer.com

Review

### The pharmacologic basis of high dose chemotherapy with haematopoietic stem cell support for solid tumours

**LF Porrata and AA Adjei**

Mayo Clinic, Division of Medical Oncology, 200 First Street SW Rochester, MN 55905, USA

**Summary** The theoretical basis of high dose chemotherapy with haematopoietic stem cell support (HDT) for solid tumours is the presumption of a linear, steep dose-response relationship for chemotherapy conditioning agents. We review preclinical pharmacologic studies evaluating steep dose-response relationships for different chemotherapeutic agents, identified through a MED-LINE and CANCER-LIT search of the English medical literature from January 1966 to December 1999. Only BCNU, melphalan, nitrogen mustard, and the combination of thiotepa and cyclophosphamide demonstrated steep dose-response relationships over a wide dose-range. The pharmacologic evidence for the use of other antineoplastic agents for HDT in solid tumours is non-existent. More preclinical studies are needed for a rational development of this therapeutic approach for solid tumours. © 2001 Cancer Research Campaign http://www.bjcancer.com

**Keywords**: preclinical pharmacology; high-dose chemotherapy; solid tumours

The modern era of chemotherapy began when patients were treated with the war gas sulfur mustard in 1931, first topically and then by direct intratumoural injection. It was however felt to be too toxic for continued human use. Nitrogen mustard, a second-generation mustard, was subsequently tested by Alfred Gilman and his colleagues, in mice and then in a patient with lymphosarcoma, in 1942. Gilman and Philips published a review of nitrogen mustard in cancer therapeutics, in 1946 (Gilman and Philips, 1946).

Since that time, conventional chemotherapy regimens have not yielded any cures in patients with most of the common epithelial neoplasms. More importantly, chemotherapy prolongs patient survival in very few metastatic solid tumours. This situation applies even to chemosensitive malignancies such as germ cell tumours and lymphomas – a significant proportion of patients are not cured by current drug regimens. Some of these patients relapse after a considerable disease-free interval, suggesting that cures may be achieved with an increase in drug dose. This idea underlies the use of high dose chemotherapy with peripheral or bone marrow stem cell rescue (HDT). HDT has led to cures of some haematological malignancies such as acute and chronic myelogenous leukemia and high-grade lymphoma (O'Donnell et al, 1987; Philip et al, 1987; Goldman, 1994). However, in solid tumors, such as breast cancer (Eddy, 1992), testicular cancer (Nichols et al, 1992), small cell lung cancer (Humblet et al, 1997), ovarian cancer (Extra et al, 1992) and sarcoma (Samuels et al, 1989), HDT has had only a minimal impact on survival (Savarese et al, 1997). The disappointing results of HDT in solid tumours have been attributed to numerous factors (Savarese et al, 1997). One of the fundamental reasons for the negative results may be faulty pharmacological assumptions. Clinical trials evaluating HDT for solid tumours have been based on the principle that anticancer agents exhibit a linear relationship between drug doses and tumour cell kill. Consequently, if toxicities associated with high doses of chemotherapy agents (up to 5-fold increased doses) could be ameliorated, then cure for solid tumours would be possible. However, the experimental evidence supporting this theory has not been rigorously reviewed. In this paper, we examine the available preclinical data to evaluate the existence of steep dose response relationships in tumour cell kill for the chemotherapy agents employed in HDT regimens.

*Received 23 November 2000*
*Revised 12 April 2001*
*Accepted 21 May 2001*

*Correspondence to:* AA Adjei

### DOSE-RESPONSE RELATIONSHIPS IN CANCER CHEMOTHERAPY

In cancer chemotherapy, a 'sleep' dose-response relationship refers to a linear relationship between drug doses and fractional cell kill for approximately 5 logs of tumour cells (Skipper et al, 1970; Jusko, 1987). The steepness of the dose-response curve implies that a disproportionately high number of cancer cells are killed when drug doses are minimally increased. This is in contrast to most other classes of therapeutic agents, which exhibit a sigmoidal dose-response relationship, with a linear relationship between dose and response over a relatively narrow range of drug doses. The plateau in this traditional curve suggests that after a certain threshold dose, further increments do not lead to an increased response (Figure 1). The concept of a steep dose response relationship for anticancer drugs dates back to the 1960s when Skipper et al (1964) predicted a log cell kill model for antineoplastic drugs. In this model, the relationship between tumour cell kill and drug dose was exponential, with the number of cells killed by a given dose of drug being proportional to both the dose of the drug and the number of cells exposed to the drug.

This model was based on observations in L1210 mouse leukemia cells, which grew exponentially until they reached a lethal tumour volume of $10^9$ cells (1 $cm^3$). Ninety-nine percent of the leukemic cells divided at a constant rate. As a result, the doubling time was always constant. In these cells, an effective

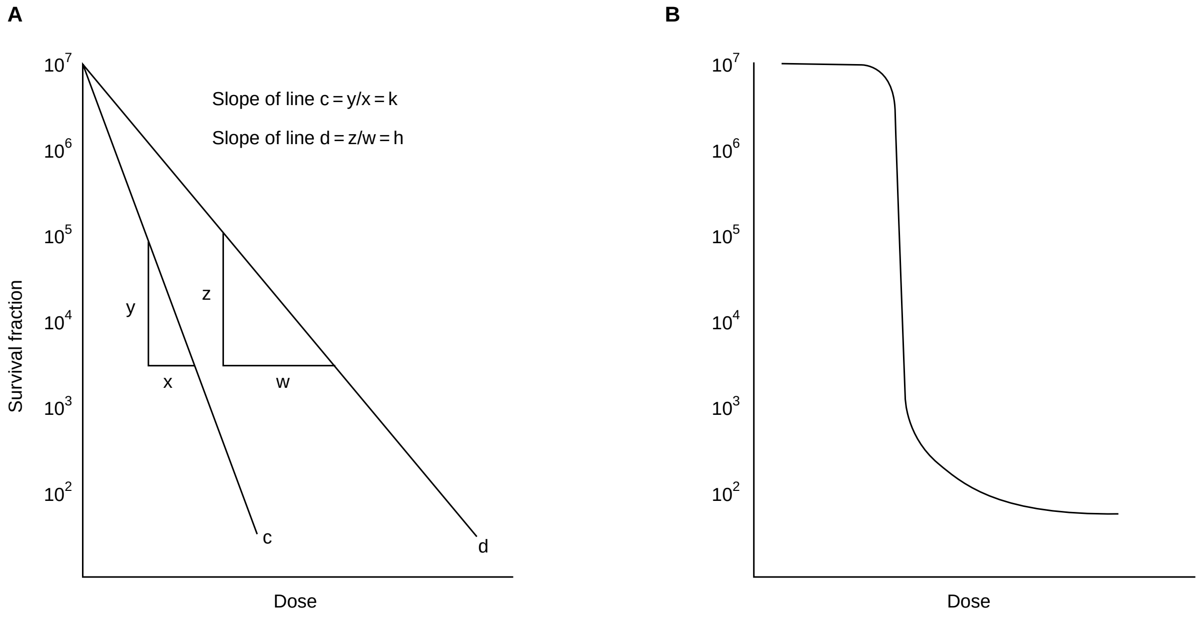

**Figure 1** Theoretical dose-response curves for anticancer agents and conventional pharmacologic agents. Graph **A** shows a linear dose-response curve demonstrating a decreased survival fraction as the drug dose increases, over a wide range of doses. Line c has a steeper slope (k) compared to line d slope (h). Both achieve the same fraction of cell kill, but at different doses. For clinical purposes, if drug d has a favourable therapeutic index, its clinical antitumour activity should not be different from drug c. Thus the linearity of the dose response curve over a broad achievable and tolerable dose-range is much more important than the steepness of the curve. Graph **B** demonstrates a dose-response curve with a plateau as drug doses are increased. Graph **A** represents the accepted nature of the dose-response curve for cytotoxic agents. Graph **B** represents the dose-response curve for most other pharmacologic agents chemotherapy regimen killed a constant fraction of exponentially growing tumour cells, regardless of the initial tumour size. If a given dose of a drug reduced $10^6$ cells to $10^5$ cells, the same therapy applied against $10^4$ cells would result in $10^3$ surviving cells. These two cytoreductions are both examples of 'one-log' kill, resulting in a 90% decrease in cell number.

Goldin et al (1956) had also demonstrated that for many drugs, the log-kill increased with increasing dose. In later studies by Frei and Canellos (1980), a log-linear relationship between drug dose and tumour cytoreduction was demonstrated in the AKR murine lymphoma as well as L1210 cells. Frei et al (1988) subsequently demonstrated a steep dose-response relationship in the MCF-7 human breast carcinoma cell line with the alkylating agents BCNU, melphalan, and nitrogen mustard. These experiments demonstrated that for many drugs, the log-kill increased with increasing dose. Moreover, if two or more sensitive chemotherapy drugs were combined, the log-kill would be multiplicative. This implied that if drug A kills 90% of the cells and a given dose of drug B kills 90%, drug A given with drug B should kill 90% of the 10% of cells left, resulting in a 99% cell kill. In this model, tumours less than $10^6$ cells should theoretically be eradicated by six cycles of a three-chemotherapy regimen.

These principles provided a rationale for adjuvant chemotherapy following removal of all clinically detectable tumour, when the remaining tumour burden could be only $10^4$ to $10^6$ cells. However, as has become evident from adjuvant studies in different epithelial tumours, the vast majority of patients are not cured. These findings have been explained by the presence of drug resistant clones among the tumour cells. To achieve cure, therefore, intrinsic and acquired drug resistance of tumour cells have to be circumvented. HDT has been one approach in the attempt to overcome drug resistance.

### DOSE INTENSITY (DI)

With the acceptance of a 'steep' dose response curve of chemotherapy agents for solid tumours, increased interest in delivering maximum tolerated drug doses as a strategy for overcoming resistance to chemotherapy emerged. Levin and Hryniuk (1987) introduced the concept of dose intensity, defined as the milligrams per square metre of delivered drug per week of therapy. In a retrospective study, these workers demonstrated that dose intensity was an independently significant correlate of relapse-free survival in women with node-positive breast cancer (Hryniuk and Lavine, 1986). In this study, there was a linear relationship between the projected increased dose intensity and improved 3-year relapse-free survival ($P < 0.00001$). Similar results were reported in another retrospective study by Levin and Hryniuk (1987) showing improved median survival time with increased dose intensity in ovarian cancer. These studies by Hryniuk and Levine triggered an interest in the investigation of dose intensity in randomized prospective controlled studies, in a wide variety of neoplasms. These studies have demonstrated that dose intense chemotherapy improves the outcome of patients with acute myelogenous leukemia, multiple myeloma, and relapsed non-Hodgkin's lymphoma (Savarese et al, 1997). However, in solid tumours, dose intense chemotherapy has yielded conflicting results (Savarese et al, 1997). A majority of studies have shown a higher response rate for the dose intense arm versus the conventional arm, but in the NSABP B-22 study (Fisher et al, 1997), a total of 2305 women with node-positive breast cancer were randomized to one of three treatment arms involving dose-escalated or dose-intensified cyclophosphamide plus adriamycin. Group 1 was defined as the standard arm (A = 60 mg/m$^2$, C = 600 mg/m$^2$ $\times$ 4 cycles); group 2 as the intensified arm (A = 60 mg/m$^2$ $\times$ 4 cycles, C = 1220 mg/m$^2$

*© 2001 Cancer Research Campaign*

× the first 2 cycles); and group 3 as the intensified and increased total dose arm (A = 60 mg/m², C = 1200 mg/m² × 4 cycles). Through 5 years of follow-up, there were no statistically significant differences in disease-free survival (DFS) or overall survival (OS) among the different groups. One explanation for the negative results in B-22 was that the doses of cyclophosphamide were too low to reveal a significant difference. To evaluate that possibility, the NSABP conducted study B-25, in which the highest dose in B-22 was used as the 'baseline' and proceeded to further escalate cyclophosphamide, again testing both cumulative dose and dose intensity (Fisher et al, 1999). All patients received growth factors. There was no improvement in DFS or OS among the three arms of the study. Several factors may explain the lack of a survival difference between dose intense regimens and conventional therapy. Tumour cell heterogeneity may affect response to dose intense regimens. Muss et al (1994) showed that dose intense therapy improved outcome among the patients in whom the tumour markedly over-expressed the c-erb2 (HER2) oncogene. Apart from molecular characteristics, tumour factors such as tumour cell kinetics, tumour size, and duration of tumour growth, as well as drug features such as dose, schedule and delivery are all likely to contribute to varying extent to determine the outcome of a particular treatment. Another possible explanation for the lack of improved survival by dose intense therapy is the concept of a threshold dose. In a study reported by Wood et al (1994), 1572 patients were divided into three groups using different doses of CAF (cyclophosphamide, adriamycin, and fluorouracil). After a median of 3.4 years of follow-up, the patients treated with high or moderate dose intensity had significant longer DFS ($P < 0.001$) and OS ($P = 0.004$) than those treated with a low dose intensity. However, when comparing the DFS and OS between the high and moderate dose intensity groups there was no significant difference ($P = 0.28$ and $P = 0.21$, respectively). These results suggest that lower threshold doses of therapy are associated with inferior results and that higher doses than the threshold dose of therapy do not affect outcome. Unfortunately, for most tumours, the threshold dose of antineoplastic agents is unknown. It is likely that the resistance mechanisms operating in solid tumours in the clinical setting cannot be totally overcome by augmenting the dose of currently available drugs. However, it can be argued that HDT may overcome resistance but simply doubling the dose intensity, as was done in most of these studies (Levin et al, 1993), is not enough.

**Table 1** Conditioning agents used in HDT for solid tumours

| | | |
|---|---|---|
| BCNU | Epirubicin | Mitoxantrone |
| Busulfan | Etoposide | Paclitaxel |
| Carboplatin | Hydroxyurea | Teniposide |
| Chlorambucil | Ifosfamide | Thiotepa |
| Cisplatin | Lomustine | Topotecan |
| Cyclophosphamide | Melphalan | Vinblastine |
| Dacarbazine | Methchlorethamine | Vincristine |
| Doxorubicin | Mitomycin C | |

**Table 2** HDT conditioning agents demonstrating a log-linear dose-response

| Drug | Cell line | Linearity of response |
|---|---|---|
| BCNU | Breast MCF-7 (H) | 5-logs |
| Melphalan | Breast MCF-7 (H) | 5-logs |
| | Fibrosarcoma FsaII (M) | 5-logs |
| | Head & Neck (H) | 5-logs |
| Nitrogen mustard | Breast MCF-7 (H) | 5-logs |
| Cyclophosphamide + Thiotepa | Breast MCF-7 (H) | 5-logs |

H = human cell line; M = murine cell line.

**Table 3** HDT conditioning agents demonstrating a narrow log-linear-dose-response

| Drug | Cell line | Linearity of response |
|---|---|---|
| Cisplatin | SCLC (H) | 2-logs |
| | Testicular SuSa (H) | 2-logs |
| | Fibrosarcoma FsaII (M) | 2-logs |
| | Lung V79-171B (M) | 2-logs |
| Cyclophosphamide | Breast MCF-7 (H) | 2-logs |
| | Fibrosarcoma FsaII (M) | 2-logs |
| Doxorubicin | Testicular SuSa (H) | 2-logs |
| Mitoxantrone | Breast (H) | 2-logs |
| | Ovarian (H) | 1-log |
| Paclitaxel | Astrocytoma U-373 (H) | 2-logs |
| | Breast MCF-7 (H) | 1-log |
| | Lung A-549 (H) | 2-logs |
| | Ovarian OVG-1 (H) | 2-logs |
| Thiotepa | Breast MCF-7 (H) | 2-logs |
| Topotecan | Breast MCF-7 (H) | 2-logs |

H = human cell line; M = murine cell line.

**Table 4** HDT conditioning agents with no reported preclinical studies

| | |
|---|---|
| Busulfan | Ifosfamide |
| Carboplatin | Lomustine |
| Chlorambucil | Mitomycin-C |
| Dacarbazine | Teniposide |
| Epirubicin | Vincristine |
| Hydroxyurea | |

### PRECLINICAL EVIDENCE FOR STEEP DOSE-RESPONSE RELATIONSHIPS IN SOLID TUMOURS

Chemotherapy agents which are commonly used as conditioning agents for HDT, are shown in Table 1. The available data from preclinical studies of dose-response relationships of these agents in solid tumour models, have been summarized in Tables 2, 3, and 4. No studies could be found testing the existence of a steep dose-response relationship for a majority of the chemotherapy agents, including busulfan, carboplatin, carmustine, chlorambucil, decarbazine, epirubicin, hydroxyurea, and ifosfamide. Preclinical studies have failed to show a log-linear dose-response relationship over a broad range of doses in solid tumours for mitoxantrone (Von Hoff et al, 1986), paclitaxel (Liebmann et al, 1993), topotecan (Ma et al, 1998), and vinblastine (Von Hoff et al, 1986). The dose response curve for these agents is curvilinear. An initial proportional tumour cell kill with dose increments, is followed by a plateau, as doses are further increased.

### Paclitaxel

Dose-response relationships for paclitaxel have been evaluated in vitro, utilizing human breast, ovary, non-small cell lung and astrocytoma cell lines. (Liebmann et al, 1993). In the MCF-7 breast cancer cell line, 5 μM of paclitaxel produced a 0.5 log cell kill,

 *© 2001 Cancer Research Campaign*

while 10 μM of drug produced a 1.5 log cell kill. However, this magnitude of cell kill remained unchanged at higher paclitaxel doses of up to 10 μM (Liebmann et al, 1993). In OVG-1 ovarian, A-549 lung, and U-373 astrocytoma cell lines, 5 μM of paclitaxel produced a 1-log cell kill. Doubling the paclitaxel dose to 10 μM led to a 2-log cell kill. Further increments in paclitaxel doses failed to increase cell kill. These results indicate a steep dose-response relationship over a narrow concentration range for paclitaxel. In these systems, there is no incremental cell kill after a concentration of 10 μM. The clinical implication would be that increasing the amount of administered drug above a dose that would achieve intra-tumoural levels above 10 μM would only increase drug toxicity with no improvement in efficacy.

Similar results were found with cisplatin, cyclophosphamide, doxorubicin and thiotepa. In all the available studies with these agents, dose-response evaluations were reported for only two-fold dose increments. It is unclear whether higher drug doses were tested and found not to yield any further tumour cell kill.

### Cisplatin

A doubling of the proportion of tumour cells killed with a doubling of the cisplatin dose was seen in a human small cell lung cancer (von Hoff et al, 1986), and testicular cancer cell line (Walker et al, 1987), as well as in the FsaII murine fibrosarcoma (Frei et al, 1988) and V79-171B chinese hamster lung cancer cell lines (Durland and Godie, 1987). Data for higher doses of cisplatin were not provided in these reports.

### Cyclophosphamide

In vitro data were found for cyclophosphamide only in the FsaII murine fibrosarcoma (Frei et al 1988) cell line. Doubling the dose of cyclophosphamide in this cell line led to a 2-fold increase in tumour cell kill. Again, data for higher doses of cyclophosphamide were not provided.

### Doxorubicin

In a similar fashion to cyclophosphamide, doubling the dose of doxorubicin from 1.25 to 2.5 ng/ml produced a 2-fold cell kill in the SuSa human testicular cancer cell line (Walker et al, 1987). Further dose increments were not reported.

### Thiotepa

Increasing the dose of thiotepa from 175 μM to 300 μM led to a 2-fold increase in cell kill in MCF-7 cells (Teicher et al, 1988). No further data was provided regarding further dose increases.

### BCNU

A clear illustration of a steep dose-response relationship over 4 logs of cell kill – a close approximation to the Skipper–Schabel model, was obtained in MCF-7 cells treated with the alkylating agent, BCNU. In this cell line, 100 μM of BCNU produced a 2-log cell kill. Doubling the BCNU dose to 200 μM led to a 4-log cell kill (Frei et al, 1988). From these in vitro studies, BCNU would be a rational choice for HDT in breast cancer.

### Melphalan

A doubling of the proportion of tumour cells killed with a doubling of the melphalan dose was seen in head and neck cancer (no cell line reported) (Von Hoff et al, 1988). In MCF-7 cells, 25 μM of melphalan produced a 2-log cell kill. Doubling the melphalan dose to 50 μM produced a 4-log cell kill. 25 mg/kg of melphalan produced a 2.5-log cell kill in FsaII murine fibrosarcoma (Frei et al, 1988). Doubling the melphalan dose to 50 mg/kg produced a 5-log cell kill. Melphalan would also be a rational choice for HDT in breast cancer.

### Nitrogen mustard

In MCF-7 cells (Frei et al, 1988), 10 μM of nitrogen mustard produced a 2.5-log cell kill, while 20 μM produced a 5-log cell kill. Nitrogen mustard would be another rational choice for HDT for breast carcinoma.

### COMBINATION STUDIES

In spite of the lack of preclinical evidence for steep dose-response relationships for some alkylating agents, some workers demonstrated that synergistic cytotoxicity of certain drug combinations achieved, in effect, steep dose-response relationships in a variety of solid tumours. Schabel et al (1978; 1984) demonstrated synergistic cytotoxicity of cyclophosphamide/nitrosourea, cyclophosphamide/melphalan, and melphalan/nitrosourea combinations in a variety of cultured and implanted murine tumors. Utilizing MCF-7 cells, Teicher et al (1988) demonstrated a steep dose-response relationship with thiotepa and cyclophosphamide. At a thiotepa dose of 50 μM and a cyclophosphamide dose of 50 μM, a 2-log cell kill was obtained. Increasing the cyclophosphamide dose to 100 μM and maintaining the thiotepa dose at 50 μM produced a 4-log of cell kill.

### CLINICAL RESULTS OF HDT IN SOLID TUMOURS

The in vitro and in vivo studies by Frei et al (1988) and Von Hoff et al (1986) discussed above as well as the studies by Schabel et al (1978; 1984) demonstrating therapeutic synergism of combined alkylating agents laid down the ground work for numerous Phase I/II studies to evaluate HDT in solid tumours. In these HDT trials, drug escalation continues until non-haematological toxicity becomes dose-limiting. Dose intensification ranged from 3-fold of standard doses (cisplatin) to over 100-fold (dacarbazine). The toxic death rates following treatment in these studies range from 0 to 14%. While these death rates may not be significantly higher than those seen in some studies utilizing standard doses of chemotherapy, the incidence of immediate and long-term morbility has not been adequately addressed.

In addition to breast, lung, ovarian, and testicular cancer, HDT has also been evaluated for colon cancer, CNS malignancies, and sarcomas. Results from these studies are sketchy and inconclusive. From the few data available, the duration of response for these tumors are 3–8 months for colon cancer, 3–10 months for CNS malignancies, and 3–66 months for sarcomas.

The clinical outcome of patients treated with HDT has been comprehensively reviewed recently (Savarese et al, 1997). In addition, four large prospectively randomized studies of HDT in breast

© 2001 Cancer Research Campaign cancer were recently reported. One study was in metastatic breast cancer and three were in high-risk breast cancer patients (10 or more positive lymph nodes and no distant metastases). In the study of HDT and conventional therapy for metastatic breast cancer, there was no significant advantage of HDT over standard therapy (Stadtmauer et al, 2000). The median survival was 24 months with HDT and 26 months with conventional CMF. Three-year survival was 32% with HDT and 38% with conventional CMF ($P$ = 0.28). Median time to progression was 9.6 months with HDT and 9 months with conventional CMF ($P$ = 0.31). An intent to treat analysis demonstrated no difference in overall survival between the two groups ($P$ = 0.52). The CALGB 9082 Study (Peters et al, 1999) for high-risk breast cancer patients, reported early results of the first 341 patients randomized to HDT versus an intermediate-dose regimen out of a total of 785 patients randomized in the study. The 3-year event-free survival was 68% in HDT group versus 64% in the intermediate dose group ($P$ = 0.1). The Scandinavian Breast Cancer Study Group 9401 for high-risk breast cancer patients reported preliminary data of 525 women randomized to nine courses of FEC (5-FU, Epirubicin, and cyclophosphamide) or three courses of FEC followed by HDT (cyclophosphamide, 6 g/m$^2$; thiotepa, 0.5 g/m$^2$; and carboplatin 0.8 g/m$^2$) (The Scandinavian Breast Cancer Study Group 9401, 1999). At a median follow-up of 23.7 months, there was no difference in the number of relapses or in overall survival. Bezwoda et al (1999) reported the only randomized study showing a significant benefit of HDT over conventional therapy in high-risk breast cancer patients. However, this study has been discredited because of suspected scientific misconduct. Overall, these randomized studies suggest that HDT may not offer any additional survival benefit compared to conventional therapy, for breast cancer. It should be noted that the HDT regimens in these studies contained agents for which we could find no proof of a steep dose-response relationship over a wide concentration range in human tumours. These agents are etoposide, mitoxantrone, carboplatin, and cisplatin.

### CONCLUSION

This review identified preclinical data supporting HDT with BCNU, melphalan, and nitrogen mustard in cultured human tumours and xenografts. Synergism was also demonstrated for combinations of cyclophosphamide and thiotepa with each other, and with the nitroso-ureas, melphalan and nitrogen mustard. A study of Table 4 indicates that there is no experimental basis for the choice of the majority of preparative regimens used in HDT for solid tumors. The choice of agents has been based on toxicity patterns. This empiricism may explain in part the lack of clinical success with HDT.

While pre-clinical models do not accurately predict clinical outcome, it may still be useful to employ such models to select agents which possess a log-linear relationship between dose and tumour cell kill over a wide range of achievable concentrations in the target tumours. In addition, specific high dose combinations may have utility in tumours with certain molecular characteristics such as HER2 over-expression. Such an approach would eliminate one confounding factor as clinical investigators try to understand the limitations of HDT. Secondly, specific hypotheses could be rationally tested.

### ACKNOWLEDGEMENTS

Supported in part by Grant CA 77112 from the National Cancer institute.

### REFERENCES

Bezwoda WR (1999) Randomized, controlled trial of high-dose chemotherapy (HD-CNVp) versus standard dose (CAF) chemotherapy for high risk, surgically treated, primary breast cancer. *Proceedings of ASCO* **18**: 2a: (abstr)

Durand RE and Goldie JH (1987) Interaction of etoposide and cisplatin in an in vitro tumor model. *Cancer Treat Rep* **71**: 673–679

Eddy DM (1992) High dose chemotherapy with autologous bone marrow transplantation for the treatment of metastatic breast cancer. *J Clin Oncol* **10**: 657–670

Extra JM, Dieras V, Giacchetti S et al (1992) High dose chemotherapy (HCT) with autologous bone marrow reinfusion (ABMR) as consolidation therapy for patients with advanced ovarian adenocarcinoma. *Proc Am Soc Clin Oncol* **11**: 234

Fisher B, Anderson S, Wickerham DL et al (1997) Increased intensification and total dose of cyclophosphamide in a doxorubicin-cyclophosphamide regimen for the treatment of primary breast cancer: findings from National Surgical Adjuvant Breast and Bowel Project B-22. *J Clin Oncol* **15**: 1858–1869

Fisher B, Anderson S, DeCillis A et al (1999) Further evaluation of intensified and increased total dose of cyclophosphamide for the treatment of primary breast cancer: findings from the National Surgical Adjuvant Breast and Bowel Project B-25. *J Clin Oncol* **17**: 3374–3388

Frei E III and Canellos GP (1980) Dose: A critical factor in cancer chemotherapy. *Am J Med* **69**: 585–594

Frei III E, Teicher BA, Holden SA, Cathcart KNS and Wang Y (1988) Preclinical studies and clinical correlation of the effect of alkylating dose. *Cancer Research* **48**: 6417–6423

Gilman A and Philips FS (1946) The biological actions and therapeutics applications of *B*-chloroethyl amines and sulfiles. *Science* **103**: 409

Goldin A, Venditti JM, Humphreys SR and Mantel N (1956) Influence of the concentration of leukemic inoculum on the effectiveness of treatment. *Science* **123**: 840

Goldman JM (1994) Management of chronic myeloid leukemia. *Blood Rev* **8**: 21–29

Hryniuk W and Levine MN (1986) Analysis of dose intensity for adjuvant chemotherapy trials in stage II breast cancer. *J Clin Oncol* **15**: 1162–1170

Humblet V, Symann M, Bosly A et al (1987) Late intensification chemotherapy with autologous bone marrow transplantation in selected small cell carcinoma of the lung: a randomized study. *J Clin Oncol* **5**: 1864–1873

Jusko WJ (1987) A pharmacodynamics of chemotherapeutic effect: dose-time-response relationships for phase nonspecific agents. *J Pharm Sci* **60**: 892–895

Levin L and Hryniuk WM (1987) Dose intensity analysis of chemotherapy regimens in ovarian carcinoma. *J Clin Oncol* **5**: 756–767

Levin L, Simon K and Hryniuk WM (1993) Importance of multiagent chemotherapy regimens in ovarian carcinoma. Dose intensity analysis. *J Natl Cancer Inst* **85**: 1732–1742

Liebmann JE, Cook JA, Lipshultz C et al (1993) Cytotoxic studies of paclitaxel (Taxol) in human cell lines. *Br J Cancer* **68**: 1104–1109

Ma J, Maliepaard M, Nooter K et al (1998) Synergistic cytotoxicity of cisplatin and topotecan or SN-28 in a panel of eight solid tumor cell lines in vitro. *Cancer Chemother Pharmacol* **41**: 307–316

Muss HB, Thor AD, Berry DA et al (1994) *c-er* B-2 expression and response to adjuvant therapy in women with noe-positive early breast cancer. *N Engl J Med* **330**: 1260–1266

Nichols CR, Andersen J, Lazarus HM et al (1992) High dose carboplatin and etoposide with autologous bone marrow transplantation in refractory germ cell cancer: an Eastern Cooperative Oncology Group Protocol. *J Clin Oncol* **10**: 558–563

O'Donnell MR, Nademonee AP, Snyder DS et al (1987) Bone marrow transplantation for myelodysplastic and myeloproliferative syndrome. *J Clin Oncol* **5**: 1822–1826

Peters WP, Rosner G, Vredenburg J et al (1999) A prospective, randomized comparison of two doses of combination alkylating agents (AA) as consolidation after CAF in high-risk primary breast cancer involving ten or more axillary lymph nodes (LN): preliminary results of CALGB 9082/SWOG 9114/NCIC MA-13. *Proceedings of ASCO* **18**: la: (abstr)

Philip T, Armitage JO, Spitzer G et al (1987) High dose therapy and autologous bone marrow transplantation after failure of conventional chemotherapy in adults with intermediate-grade or high-grade non-Hodgkin's lymphoma. *N Engl J Med* **316**: 1493–1498

Samuels B, Eilas A, Vogelzang N et al (1989) High dose chemotherapy with autologous bone marrow reinfusion for refractory sarcomas. *Proc Am Assoc Cancer Res* **30**: 273

© 2001 Cancer Research Campaign

Savarese DMF, Hsiech CC and Stewart FM (1997) Clinical impact of chemotherapy dose escalation in patients with hematologic malignancies and solid tumors. *J Clin Oncol* **15**: 2981–2995

Schabel FN Jr, Trader NW, Laster WR Jr et al (1978) Patterns of resistance and therapeutic synergism among alkylating agents. *Antibiot Chemother* **23**: 200–215

Schabel FN Jr, Griswold DP Jr and Corbit TH (1984) Increasing therapeutic response rates to anticancer drugs by applying the basic principles of pharmacology. *Pharmacol Therapeut* **20**: 282

Skipper HE, Schabel FM Jr and Wilcox WS (1964) Experimental evaluation of potential anti-cancer agents XIII: on criteria and kinetics associated with “curability” of experimental leukemia. *Cancer Chemother Rep* **35**: 1

Skipper HE, Schabel FM, Mellett LB et al (1970) Implications of biochemical, cytokinetic, pharmacologic, and toxicologic relationships in the design of optimal therapeutic schedules. *Cancer Chemother Rep* **54**: 431–450

Stadtmauer EA, O’Neill A, Goldstein LJ et al (2000) Conventional-dose chemotherapy compared with high-dose chemotherapy plus autologous hematopoietic stem cell transplantation for metastatic breast cancer. *New Engl J Med* **342**: 1069–1076

Teicher BA, Holden SA, Cucchi CA et al (1988) Combination of *N*, *N*’, *N*’-Triethyenethiophosphoramide and cyclophosphamide in vitro and in vivo. *Cancer Research* **48**: 94–100

The Scandinavian Breast Cancer Study Group 9401 (1999) Results from a randomized adjuvant breast cancer study with high dose chemotherapy with $CTC_b$ supported by autologous bone marrow stem cells versus dose escalated and tailored FEC therapy. *Proceedings of ASCO* **18**: 2a: (abstr)

Von Hoff DP, Clark GM, Weiss GR et al (1986) Use of in vivo dose response effect to select antineoplastics for high-dose or regional administration regimens. *J Clin Oncol* **4**: 1827–1834

Walker MC, Paris CN and Masters JR (1987) Differential sensitivities of human testicular and bladder tumor cell lines to chemotherapy drugs. *JNCI* **79**: 213–216

Wood WC, Budman DR, Korzun AH et al (1994) Dose and dose intensity of adjuvant chemotherapy for stage II, node positive breast carcinoma. *N Engl J Med* **330**: 1253–1259

© 2001 Cancer Research Campaign
